# Synergistic Effect between Usnic Acid and Polymyxin B against Resistant Clinical Isolates of *Pseudomonas aeruginosa*

**DOI:** 10.1155/2020/9852145

**Published:** 2020-08-12

**Authors:** Sérgio Dias da Costa Júnior, Wagner Roberto Cirilo da Silva, Adriana Maria Costa Marques da Silva, Maria Amélia Vieira Maciel, Isabella Macário Ferro Cavalcanti

**Affiliations:** ^1^Laboratory of Immunopathology Keizo Asami, Federal University of Pernambuco, Recife, PE, Brazil; ^2^Department of Tropical Medicine, Federal University of Pernambuco, Recife, PE, Brazil

## Abstract

The present study aimed to characterize the susceptibility profile of *Pseudomonas aeruginosa* and *Acinetobacter* spp. clinical isolates to polymyxin B in a public hospital in Recife-PE, Brazil, between the years of 2018 and 2019, as well as to search for the presence of the *mcr*-1 gene and evaluate the interaction between polymyxin B and usnic acid against these isolates. The strains were identified using the BD Phoenix™ automated system and the agar-spot test was used to determine the susceptibility profile to polymyxin B. The minimum inhibitory concentrations (MICs) of usnic acid and polymyxin B were determined through the broth microdilution method according to the Clinical and Laboratory Standards Institute (CLSI). Subsequently, Polymerase Chain Reaction (PCR) was performed to detect the *mcr*-1 gene in the isolates. The interaction between usnic acid and polymyxin B was evaluated by the Checkerboard assay. Among 34 isolates of *P. aeruginosa*, 26.5% (9/34) were positive for the polymyxin B agar-spot test, and 11.8% (4/34) presented an intermediate susceptibility (MIC = 4 *μ*g/mL), while 14.7% (5/34) presented antimicrobial resistance with MIC values ranging from 8 to 32 *μ*g/mL. Among 38 isolates of *Acinetobacter* spp., 13.2% (5/38) were positive for the polymyxin B agar-spot test and all of them were resistant to polymyxin B with a MIC value > 32 *μ*g/mL. The *mcr*-1 gene was not detected in the clinical isolates. Regarding usnic acid, it presented a moderate antibacterial activity against two *P. aeruginosa* isolates (MIC = 250 *μ*g/mL) and no activity was detected against the others. A synergistic effect between usnic acid and polymyxin B was observed against three clinical isolates of *P. aeruginosa* which were resistant to polymyxin B (FICI ≤ 0.5). Therefore, it was possible to observe that usnic acid is a promising candidate to be used in combination with polymyxin B against infections caused by resistant *P. aeruginosa*.

## 1. Introduction

*Pseudomonas aeruginosa* and *Acinetobacter* spp. are nonfermenting Gram-negative bacilli (NFGNB) implicated with high rates of colonization and infection in hospitalized individuals who live, in their majority, in developing countries. These microorganisms are mainly associated with cases of pneumonia and bacteremia, as well as infections related to urinary tract, wounds, and catheter. Besides, they are included in a group composed of the six most prevalent multi-resistant pathogens in nosocomial infections, known as the acronym ESKAPE—*Enterococcus faecium*, *Staphylococcus aureus, Klebsiella pneumoniae*, *Acinetobacter* spp., *Pseudomonas aeruginosa*, and *Enterobacter* spp. [1, 2].

The wide variety of virulence and resistance mechanisms presented by *P. aeruginosa* and *Acinetobacter* spp. isolates, as well as their capacity of interacting and exchanging genetic material with other bacteria, stimulates the search for resistance genes in isolates found in hospital environments [2–4]. Besides, the increasing number of infections caused by multidrug-resistant (MDR) Gram-negative strains led to the return of the polymyxins (polymyxin B and colistin) in medical clinics as one of the treatment options [5, 6].

Polymyxins are polypeptide antibiotics which destabilize lipopolysaccharides (LPS) from the cell membranes of Gram-negative bacteria, resulting in a permeability increase and consequently in the externalization of cytoplasmatic contents and cell death [7]. However, in the past years, some Gram-negative species which were previously described as susceptible to polymyxins have been reported as resistant to this class of antimicrobials [2, 6, 8].

Studies have demonstrated that the resistance to polymyxins could be either intrinsic, such as those observed in pathogens like *Brucella* spp., *Burkholderia cepacia* complex, *Edwardsiella* spp., *Morganella morganii*, *Proteus* spp., *Providencia* spp. and *Serratia* spp., or acquired through chromosomal mutations. It was thereafter identified that the resistance to polymyxins was associated with a gene that could be present in mobile genetic elements, the *mcr*, encoded in a plasmid from a strain of *Escherichia coli* in China [5, 7]. In the following years, studies identified the presence of the *mcr*-1 gene and its variants in *Acinetobacter* spp. and *Pseudomonas aeruginosa* [6, 8]. In addition to that, the first cases of human infections caused by *E. coli* strains harboring *mcr*-1 genes were detected in Latin America countries, such as Brazil, Argentina, and Ecuador [9–11]. Due to the increase of MDR bacilli, the scientific community has been studying combining therapies as an option to treat diseases caused by these microorganisms. Combined therapies are used in order to get a higher potency and consequently therapeutic success of a given antimicrobial. Natural compounds can act in synergy with drugs used in medical practice allowing the reduction of the required dose to achieve the cure and minimizing side effects [12–14].

Usnic acid is one of the compounds that have been studied in the past years. It is present in different species of lichens as a product of the secondary metabolism. A series of *in vitro* studies demonstrated not only anti-inflammatory, antioxidant, antitumor, and antiviral properties, but also a potent antibacterial activity against mycobacteria and several Gram-positive and Gram-negative bacteria [15–18]. Along with these data, the interest in this substance has risen since it could be used as a potential antimicrobial compound. The mechanism of action of usnic acid has not been fully elucidated yet. However, studies have reported that this molecule interferes with the synthesis and replication of bacterial nucleic acids [19]. Therefore, the present study aimed to characterize the susceptibility profile of *P. aeruginosa* and *Acinetobacter* spp. isolates to polymyxin B in a public hospital in Recife-PE, Brazil, between the years of 2018 and 2019 and also to search for the presence of the *mcr*-1 gene and evaluate the *in vitro* interaction between polymyxin B and usnic acid against these isolates.

## 2. Material and Methods

### 2.1. Study Population

The strains analyzed in this study were collected from individuals who were admitted in a public hospital in Recife-PE, Brazil. The isolates (*n* = 72) were harvested from different regions of 72 infected or colonized patients by *P. aeruginosa* and *Acinetobacter* spp. These isolates were provided by the Bacteriology Department during the years of 2018 and 2019 and are part of a project approved by the Ethics Committee for Human Research of the Federal University of Pernambuco (CAE: 0490.0.172.000-11).

### 2.2. Clinical Isolates

The identification of the isolates was performed through the Phoenix B™ automated system. Then, the isolates were sent to the Laboratory of Bacteriology and Molecular Biology and conserved in glycerol at −20°C. These bacteria were reactivated in tubes containing brain-heart infusion (BHI) broth (HiMedia®, Mumbai, India), in which they were incubated for 48 hours at 37°C. The bacteria were then seeded in plates containing cetrimide agar and MacConkey medium, being incubated at 35 ± 2°C for 24 hours so that the purity of the colonies could be analyzed [20].

### 2.3. Polymyxin B Agar-Spot Test

Initially, a stock solution of 60 *μ*g/mL polymyxin B sulfate salt (Sigma-Aldrich®, United States) was prepared. Then, 1 mL of the stock solution was added to 19 mL of Cation-Adjusted Müeller-Hinton Broth (CAMHB) (HiMedia®, Mumbai, India), corresponding to a final concentration of 3 *μ*g/mL, at an approximate temperature of 50°C, being thus poured onto a sterile Petri dish. A bacterial suspension adjusted to 0.5 on the McFarland Scale was prepared to obtain a final concentration of 2–5 × 10^5^ UFC/well; then, it was seeded in plate as a “*spot*” and incubated at 35 ± 2°C for 20 hours. As controls, the strains *Escherichia* coli ATCC 25922 (polymyxin/colistin susceptible), *P. aeruginosa* ATCC 27853 (polymyxin/colistin susceptible), and *E. coli* NCTC 13846 (polymyxin/colistin-resistant) were used in all plates. All samples were tested in duplicate. Reading was performed after 20 h according to the following interpretation: positive growth (resistant to polymyxin B) when more than 1 colony was observed; negative growth (susceptible to polymyxin B) when there were no bacterial colonies [21]. The procedures were performed in duplicate and in two independent experiments.

### 2.4. Broth Microdilution

In order to determine the Minimal Inhibitory Concentration (MIC), 95 *μ*L of CAMHB medium was added in each well of 96-well plates. Serial dilutions of Polymyxin B ranging from 0.06 to 32 *μ*g/mL and of usnic acid ranging from 1 to 500 *μ*g/mL were added. Finally, the bacterial suspensions were adjusted to 0.5 on the McFarland scale, diluted, and deposited in the wells at a final concentration of 2–5 × 10^5^ UFC/well. The plates were then incubated at 35 ± 2°C for 20 to 24 hours [20]. As controls, the strains *E. coli* ATCC 25922, *P. aeruginosa* ATCC 27853, and *E. coli* NCTC 13846 were used. All samples were tested in triplicate using resazurin and a viability dye. The plates were read using an ELISA plate reader at 620 nm wavelength (Multiskan FC, Thermo Scientific) [22]. After incubation, 25 *μ*L of resazurin was added to each well and the plates were incubated for 1 hour at 35 ± 2°C. When color changes were observed in the wells, where a light blue turned to a bright pink, the assay was considered positive for bacterial growth. MICs values were interpreted according to the CLSI Guidelines [20].

### 2.5. DNA Extraction

After incubating the colonies in Luria Bertani (LB) medium (HiMedia®, Mumbai, India) at 35°C for 24 hours, the DNA extraction of the isolates was performed using the Brazol kit (LGC-Biotechnology, São Paulo, Brazil), according to the manufacturer's instructions. The extracted DNA was quantified by spectrophotometry using Nanodrop™ 2000/2000c (Thermo Scientific, United States), in a wavelength ranging from 260 to 280 nm.

### 2.6. Polymerase Chain Reaction (PCR)

The amplification reactions of the *mcr-1* gene used the following primers: CLR5-F [5′-CGGTCAGTCCGTTTGTTC-3′] and CLR5-R [5′-CTTGGTCGGTCTGTA GGG-3′], described by Liu et al. [5]. Each reaction included 10 ng of total DNA (1 *μ*L), 10 pmol of each primer, 200 dNTP, 1x buffer, 2.5 mM of MgCl_2_, and 1 U of Taq DNA polymerase. For each amplification, the following conditions were used: initial denaturation at 94°C for 5 minutes; 35 cycles of 1 minute at 94°C, 1 minute on the annealing temperature at 53°C, and 1 minute and 30 seconds at 72°C; followed by a final extension of 10 minutes at 72°C [23]. As the positive control, the DNA of the *E. coli* strain NCTC 13846 was included and as the negative control, ultrapure water. The PCR products were submitted to electrophoresis using a 1.5% agarose gel in 0.5x TBE buffer. These products were stained with blue-green (LGC-Biotechnology, São Paulo, Brazil) and visualized in an ultraviolet transilluminator and photo-documenter (Kasvi®).

### 2.7. Checkerboard Method

To evaluate the interaction between usnic acid and polymyxin B (Sigma-Aldrich^®^, United States) against polymyxin B-resistant *P. aeruginosa* clinical isolates, the Checkerboard method was performed [24]. Concentrated solutions of polymyxin B and usnic acid of 8 × MIC and 4 × MIC, respectively, were used. Initially, 95 *μ*L of CAMHB was added to each well of 96-well plates. In order to obtain the final MIC and dilutions with lower values than the MIC of the respective compounds, polymyxin B was added on the *X*-axis, and usnic acid on the *Y*-axis. Finally, the bacterial concentration was adjusted to a 0.5 density on the McFarland scale to obtain the final concentration of 1 to 2 × 10^5^ UFC/well. Wells of column 11 were used as growth controls, containing only the culture medium and the inoculum, whereas the wells of column 12 were used as sterile controls, containing only the culture medium. The microplates were incubated at 35 ± 2°C for 24 hours and after this period, reading was performed at 620 nm.

In order to classify the interaction between the compounds, the fractional inhibitory concentration index (FICI) was calculated according to the equation ΣFICI=FICI_*a*_+FICI_*b*_=(*C*_*a*_/MIC_*a*_)+(*C*_*b*_/MIC_*b*_), where *C*_*b*_ and *C*_*b*_ are the MIC values of the combined compounds and MIC_a_ and MIC_b_ are the MIC of the isolated compounds. The values obtained in the FICI equation were interpreted according to the classification proposed by Sopirala et al. [24]: synergistic (FICI ≤ 0.5), additive (0.5 > FICI ≤ 1.0), indifferent (1 > FICI ≤ 4.0), and antagonistic (FICI > 4.0).

## 3. Results and Discussion

### 3.1. Clinical Isolates

**Figure 1:**
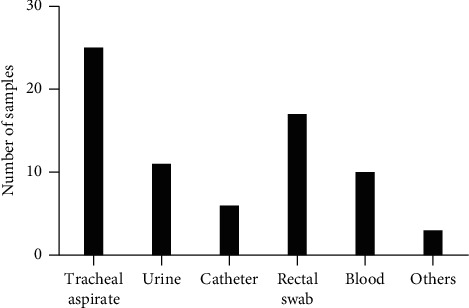
Frequency distribution of nonfermenting Gram-negative bacilli isolates in biological samples of patients from a public hospital in Recife-PE, Brazil, collected between 2018 and 2019.

Among 72 clinical isolates of NFGNB collected in a public hospital in Recife-PE, Brazil, 47.2% (34/72) were identified as *P. aeruginosa* and 52.8% (38/72) as *Acinetobacter* spp. Also, 34.7% (25/72) of the analyzed samples were from tracheal secretion, 23.6% (17/72) from rectal swab, 15.2% (11/72) from urine, 13.9% (10/72) from blood, 8.3% (6/72) from catheter tips, and 4.16% (3/72) from other body fluids. Similarly, studies demonstrate that samples from tracheal secretion, rectal swab, and urine of hospitalized patients present high frequencies of NFGNB related to colonization or infection (Figure 1) [2, 6, 25].

*P. aeruginosa* and *Acinetobacter baumannii* are the two most common NFGNB in nosocomial infections. Over the last 20 years, the numbers of multidrug-resistant bacteria from these species have gradually increased around the world, especially in developing countries [25]. Deliberali et al. [25] identified NFGNB in 326 samples from patients admitted in a hospital in Porto Alegre-MG, Brazil. Among these samples, *P. aeruginosa* was identified in 65% of the isolates and *A. baumannii* in 16.6%. The biological samples in which the higher positivity to these microorganisms was observed were tracheal aspirate (38.3%), sputum (18.7%), and urine (16%).

### 3.2. Susceptibility Profile to Polymyxin B

The increasing number of studies which report the alarming amounts of polymyxin B-resistant Gram-negative bacteria from clinical isolates stimulates the scientific community to develop new low-cost and rapid methods that can be used for clinical diagnosis [25, 26]. In the present study, 26.4% of 34 *P. aeruginosa* isolates (9/34) were positive for the polymyxin B agar-spot test presenting more than one bacterial colony, whereas among 38 isolates of *Acinetobacter* spp., 13.2% (5/38) were positive.

Regarding the susceptibility analysis to polymyxin B using the broth microdilution assay, it was observed that 11.8% (4/34) of *P. aeruginosa* isolates presented an intermediate susceptibility to polymyxin B (MIC = 4 *μ*g/mL), whereas 14.7% (5/34) presented resistance to this agent (MIC = 8 to 32 *μ*g/mL). Among the strains of *Acinetobacter* spp., 13.2% (5/38) were resistant to polymyxin B (MIC ≥ 32 *μ*g/mL). Overall, 13.9% (10/72) of the analyzed isolates were resistant to polymyxin B (Table 1).

Clinical isolates of *P. aeruginosa* and *Acinetobacter* spp. which are resistant or have a low susceptibility to polymyxin B have already been reported. The polymyxins are usually the last monotherapy option to treat infections caused by *P. aeruginosa* and *Acinetobacter* spp. strains. Isolates from these resistant species are known as pan-resistant [6, 8].

It was possible to observe 100% of similarity between the polymyxin B agar-spot test and broth microdilution assay when the susceptibility profile of the clinical isolates was characterized. The results of the present study demonstrate that the polymyxin B agar-spot test can be used as a reliable tool to screen resistant or low-susceptible *P. aeruginosa* and *Acinetobacter* spp. strains. Besides that, it can be easily implemented, and it has a low cost in a clinical laboratory setup [21].

Previous studies describe the benefits of using agar-spot tests with colistin or polymyxin B to screen samples and detect strains that are resistant to these antimicrobial agents. Fernandes et al. [27] screened 4620 isolates of enterobacteria using MacConkey agar supplemented with 2 mg/L colistin and confirmed the presence of resistant strains using the broth microdilution assay. Both tests were compatible regarding the susceptibility profile to colistin among the tested isolates. Therefore, the use of a method using supplemented media with polymyxins can facilitate the screening of a high number of samples [27].

Studies developed in Brazil between 2009 and 2017 reported that drugs such as the polymyxins should be the last therapeutic resource against multidrug-resistant strains of *P. aeruginosa* and *Acinetobacter* spp. Besides, pan-resistant strains of these same species have been found in clinical isolates from patients admitted in intensive care units (ICUs) in Brazilian hospitals [3, 28, 29].

Genteluci et al. [28] have reported an alarming frequency of polymyxin B-resistant strains of *A. baumannii* in clinical isolates from a tertiary hospital in Rio de Janeiro, Brazil. From 92 samples, 85 presented MIC values ranging from 4 to 64 *μ*g/mL and were characterized as resistant to polymyxin B. Differently from Genteluci et al. [28], a study which analyzed the activities of the polymyxins B and E against 100 clinical isolates of *P. aeruginosa* (*n* = 65) and *A. baumannii* (*n* = 35) collected from two public hospitals from Recife-PE, Brazil, demonstrated that all isolates were sensitive to both polymyxins [2].

Grewal et al. [30] investigated the presence of NFGNB among patients admitted in a tertiary care hospital in Patiala, India, between the years of 2015 and 2016. Among 1854 culture-positive samples, 216 (11.6%) comprised NFGNB. *P. aeruginosa* was the most common NFGNB, being isolated in 190 out of 216 samples (87.96%), followed by *A. baumannii* 17/216 (7.87%). Besides that, all isolates of *P. aeruginosa* were susceptible to polymyxin B, whereas only 3/17 (17.6%) of *A. baumannii* isolates were also susceptible. This data demonstrates an alarming percentage of infections caused by polymyxin B-resistant strains of *A. baumannii* in patients admitted in this hospital. A similar study evaluated the resistance profile of NFGNB present in patients admitted at the University Hospital of Nepal, between January and December 2017. Among the 1486 culture-positive cases, 402 NFGNB isolates were identified. *A. baumannii* was identified in 177/402 (44%), while *P. aeruginosa* was isolated in 161/402 (40.1%). In addition to that, all isolates of *A. baumannii* and *P. aeruginosa* demonstrated susceptibility to polymyxin B and colistin [31]. Studies conducted by Grewal et al. [30] and Yadav et al. [32] obtained different incidence results of polymyxin B-resistant *Acinetobacter* spp. and *P. aeruginosa* isolates compared to the present study. The presence of different percentages of polymyxin-resistant strains in different parts of the world is related to the public health characteristics, number of inhabitants, sanitation, and economic development, those being considered determinant factors in the health-disease process [28–30, 32].

Clinical isolates of *P. aeruginosa* which presented an intermediate susceptibility to polymyxin B were present in tracheal secretion samples, whereas resistant isolates were found in urine (3/5), catheter tip (1/5), and ulcer secretion (1/5). Polymyxin B-resistant *Acinetobacter* spp. isolates were found in tracheal aspirate (2/5), rectal swab (2/5), and urine (1/5). Similarly, a study in Pakistan using NFGNB strains has described colistin-resistant isolates of *P. aeruginosa* and *A. baumannii* in urine, wound secretion, and blood samples [8]. Both studies demonstrate the diversified prevalence in biological samples in which strains of NFGNB resistant to polymyxins can be detected.

### 3.3. Presence of the mcr-1 Gene

**Figure 2:**
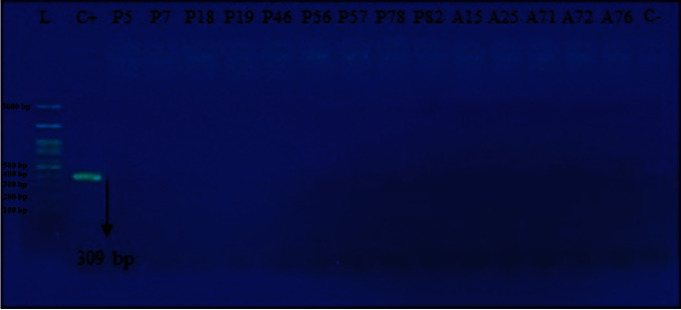
Electrophoresis of the *mcr-*1 PCR product in *Pseudomonas aeruginosa* and *Acinetobacter* spp. isolates obtained in a public hospital from Recife-PE, Brazil, collected between 2018 and 2019. L: molecular weight marker; C+: positive control (*E. coli* strain NCTC 13846); C−: negative control; bp: base pairs.

In this study, among all the polymyxin B-resistant clinical isolates, none of them presented the *mcr*-1 gene (Figure 2). This suggests that the resistance to polymyxin B in these microorganisms is related to different resistance mechanisms or even to a variation of the *mcr* gene. A study performed in Pakistan investigated the susceptibility profile to colistin in 146 clinical isolates of *A. baumannii* (*n* = 62) and *P. aeruginosa* (*n* = 84) collected in the four biggest tertiary hospitals in Peshawar. It was verified that 9.6% (6/62) of *A. baumannii* isolates and 11.9% (10/84) of *P. aeruginosa* strains were resistant to colistin. The search for the *mcr*-1 gene among colistin-resistant isolates has detected one isolate of *A. baumannii* and one of *P. aeruginosa*, being the first case of *A. baumannii* and *P. aeruginosa* strains carrying the *mcr*-1 gene [8].

The resistance to polymyxin might occur through mutations or adaptation processes or during the acquisition of plasmids that carry resistance genes. However, regarding *P. aeruginosa* and *Acinetobacter* spp., this resistance is mainly related to complex networks involved with LPS composition or triggered by chromosomal mutations and/or differences in gene expression levels related to the LPS components. The present study did not detect the *mcr-*1 gene in NFGNB, and this fact demonstrates that these isolates might have acquired resistance through mutations or bacterial adaptation. Studies emphasize that research involving polymyxin resistance mediated by plasmids in NFGNB is needed mainly due to the constant variation in molecular epidemiology and because this gene has already been described in NFGNB isolates in other countries, such as Iraq [8, 26].

### 3.4. Usnic Acid Antibacterial Activity

During the evaluation of its antibacterial activity, it was observed that usnic acid was not effective against *Acinetobacter* spp. Isolates; however, it presented an inhibitory activity in all isolates of *P. aeruginosa* (MIC = 250 and 500 *μ*g/mL) (Table 2). The results obtained in the present work corroborate with the literature. In a study performed by Maciąg-Dorszyńska et al. [33], usnic acid presented antibacterial activity against Gram-positive bacteria such as *Bacillus subtilis* and *Staphylococcus aureus*; however, when evaluated for *Escherichia coli*, no activity was observed.

In a study conducted by Francolini et al. [31], the isomer (+)-usnic acid presented a MIC of 32 *μ*g/mL against *S. aureus*, whereas for *P. aeruginosa*, its MIC was 256 *μ*g/mL. Ranković et al. [34] evaluated the antimicrobial activities of an acetonic extract from *Usnea barbata* and usnic acid purified from the same lichen species against Gram-positive microorganisms such as *B. subtilis*, *B. mycoides*, and *S. aureus* and the Gram-negative bacilli *E. coli* and *K. pneumoniae*. The acetonic extract and usnic acid presented MIC values of 250 *μ*g/mL and 500 *μ*g/mL, respectively, against the Gram-positive bacteria. When evaluated against the Gram-negative bacteria, the extract and usnic acid presented MIC values of 125 *μ*g/mL and 500 *μ*g/mL, respectively.

### 3.5. Synergistic Antimicrobial Activity of Polymyxin B and Usnic Acid

According to research performed on pharmacokinetics (PK), pharmacodynamics (PD), and toxicity and also from clinical trials using polymyxin B and colistin, these agents have a limited clinical utility. Data regarding PK and PD report a bactericidal concentration around 2 *μ*g/mL. However, most patients with renal insufficiency cannot reach this dosage, and the use of these antimicrobials is also associated with nephrotoxicity. Two randomized clinical trials have evaluated the mortality rate in patients infected by Gram-negative bacteria resistant to carbapenems and identified a 30% to 40% mortality associated with colistin in 28 days of treatment, whereas the mortality rates associated with the use of imipenem-relebactam and plazomicin were 10 and 12%, respectively [35, 36]. Therefore, the need for studies regarding alternative treatments is urgent [2, 4, 37].

The emergence of infections caused by resistant bacteria has encouraged the use of combined therapeutic approaches in the medical clinic. The results of the combined use of piperacillin-tazobactam associated with tobramycin, as well as ceftazidime with tobramycin, had satisfactory rates of synergistic activity against MDR *P. aeruginosa* strains. In recent years, the use of cephalosporins associated with *β*-lactamase inhibitors has evolved with the emergence of new options for *β*-lactamase inhibitors. Ceftolozane-tazobactam and ceftazidime-avibactam were introduced as therapeutic options for the treatment of infections caused by MDR and extensively drug-resistant (XDR) *P. aeruginosa*, but these antibiotics presented a low inhibitory activity against metallo-*β*-lactamase (MBL)-producing *P. aeruginosa*. The use of aztreonam-avibactam, on the other hand, had a satisfactory activity when evaluated against enterobacteria and MBL-producing *P. aeruginosa*. Recently, the use of minocycline and doxycycline has been indicated for infections caused by MDR or XDR *Acinetobacter* spp. [38, 39].

In the present study, the association between polymyxin B and usnic acid against polymyxin-resistant *P. aeruginosa* clinical isolates was evaluated through the *Checkerboard* method since usnic acid alone did not present any antibacterial activity against *A. baumannii* isolates (MIC > 500 *μ*g/mL). The FICI observed in the association between usnic acid and polymyxin B varied between 0.0625 and 0.156, being classified as synergistic for all polymyxin B-resistant *P. aeruginosa* isolates (FICI ≤ 0.5) (Table 3). According to a study from Ferraz-Carvalho et al. [15], the combination of usnic acid with rifampicin has been synergistic against *Mycobacterium tuberculosis* isolates. Therefore, due to its recognized ability to increase the therapeutic potential of commercial drugs, usnic acid has been used as a promising alternative against resistant clinical isolates.

There are limitations regarding the use of usnic acid in *in vivo* models of systemic infections, due to its low aqueous solubility and hepatotoxicity after systemic administration [40]. Therefore, investigations on the therapeutic use of usnic acid *in vivo* have been focused on the topical application of this compound, especially in the treatment of infected wounds [19, 40, 41]. The study conducted by Zhang et al. [41] demonstrated through histopathological analyses that usnic acid had a healing effect in rats after its topical application. In this study, the following was observed: decrease in inflammatory cells, vascular regeneration, increase of fibroblast proliferation, early re-epithelialization, and epidermal keratinization.

An alternative strategy to reduce usnic acid's side effects is its encapsulation into drug delivery systems [40]. Some studies have already proposed the encapsulation of the acid into liposomes and evaluated its potential in *in vitro* experiments. Research conducted by Nunes et al. [42] investigated the possible curative effects of Lipo-usnic acid and gelatin-based films on epidermis wounds caused by burns using an *in vivo* swine model. After treatment with Lipo-usnic acid, tissue analyses have demonstrated the development and maturation of granulation tissue and repair of scars with the presence of satisfactory collagen deposition. The suggested mechanism of action of Lipo-usnic acid might be through its ability of promoting cellular motility. In addition, there was no presence of bacterial infections in these animal wounds [42]. However, no *in vivo* studies investigating the activity of usnic acid or usnic acid encapsulated in liposomes in systemic infections have been carried out yet. We also emphasize the importance of our results for future *in vivo* studies that aim to investigate the interaction between usnic acid and antibiotics, since the *in vitro* doses of these drugs are reduced and may reflect the possibility of using this combination in systemic studies.

On synergistic combinations, the involved compounds can act under different pharmacological targets neutralizing resistance mechanisms and eliminating side effects, also interacting with each other increasing their solubility and bioavailability [43]. Only a few studies suggest the possible antibacterial mechanism of action of usnic acid [19]. Gupta et al. [44] have demonstrated that usnic acid acts against *S. aureus* by increasing its membrane permeability leading to cell lysis. Maciąg-Dorszyńska et al. [33] verified that usnic acid can also cause the inhibition of RNA and DNA synthesis in some bacteria. In a more recent study, Sinha et al. [45] demonstrated that usnic acid acts in synergy with norfloxacin against *S. aureus* by inhibiting efflux pumps, inducing oxidative stress, inhibiting fatty acids and peptidoglycans production, altering thus the membrane potential and the metabolic activity of the bacteria destabilizing its cellular membrane.

Therefore, it is suggested that usnic acid favors the destabilization of the bacterial membrane in the presence of polymyxin B, intensifying the electrostatic interaction with lipid A. Currently, only a few antimicrobial agents are under development; what causes concern the scientific community is the emergence of resistance mechanisms that are being constantly reported around the world. Thus, research on new therapies is of extreme importance especially regarding resistant strains of *P. aeruginosa*, for this microorganism is the most commonly involved pathogen in infected burns and wounds, as well as in systemic infections of hospitalized patients [3, 13].

The perspectives of this study include performing *in vivo* experiments to evaluate the combination between usnic acid and polymyxin B for the treatment of wound infections, and other superficial or systemic infections caused by polymyxin B-resistant *P. aeruginosa*, as well as the encapsulation of usnic acid into liposomes and the evaluation of the *in vivo* antibacterial activity of this Lipo-usnic acid with polymyxin B.

## 4. Conclusion

In the present study, it was possible to detect an alarming frequency of polymyxin B-resistant strains of *P. aeruginosa* and *Acinetobacter* spp. in patients from a public hospital in Recife-PE, Brazil. Besides, until now, no strains of these microorganisms harboring the *mcr* gene have been reported in Brazil. Thus, we reinforce the importance of local studies including phenotypic characterization and molecular epidemiology in different time intervals. Through these studies, nosocomial infections can be monitored in different hospital units, avoiding the dissemination of pathogenic microorganisms with resistant phenotypes. With that, the scientific community will be aware of the need of new therapeutic options and the rational use of antimicrobial agents, therefore assisting in the fight against bacterial resistance. The importance of detecting new genes and variants of the *mcr* gene, as well as the construction of dissemination data on resistant NFGNB, is also highlighted. The data analysis regarding the antibacterial activity of usnic acid as a monotherapy demonstrated that this agent is weak or moderately effective against clinical isolates of *Pseudomonas aeruginosa* and *Acinetobacter* spp. However, in combination with polymyxin B, usnic acid had a synergistic effect against polymyxin B-resistant *P. aeruginosa* clinical isolates. Therefore, usnic acid is a promising candidate against infections caused by resistant *P. aeruginosa* strains when combined with polymyxin B.

## Figures and Tables

**Table 1:** Susceptibility profile to polymyxin B of *Pseudomonas aeruginosa* and *Acinetobacter* spp. clinical isolates.

Clinical isolates	Identification of the bacteria	Polymyxin B agar-spot test	MIC (*μ*g/mL)
A15	*Acinetobacter* spp.	+	>32 [R]
A25	*Acinetobacter* spp.	+	>32 [R]
A71	*Acinetobacter* spp.	+	>32 [R]
A72	*Acinetobacter* spp.	+	>32 [R]
A76	*Acinetobacter* spp.	+	>32 [R]
PA05	*Pseudomonas aeruginosa*	+	4 [I]
PA07	*Pseudomonas aeruginosa*	+	4 [I]
PA18	*Pseudomonas aeruginosa*	+	8 [R]
PA19	*Pseudomonas aeruginosa*	+	4 [I]
PA46	*Pseudomonas aeruginosa*	+	32 [R]
PA56	*Pseudomonas aeruginosa*	+	8 [R]
PA57	*Pseudomonas aeruginosa*	+	32 [R]
PA78	*Pseudomonas aeruginosa*	+	32 [R]
PA82	*Pseudomonas aeruginosa*	+	4 [I]

MIC: minimum inhibitory concentration; R: resistance; I: intermediate resistance.

**Table 2:** Antimicrobial activity of usnic acid against clinical isolates of *Acinetobacter baumannii* and *Pseudomonas aeruginosa*.

Clinical isolates	Identification of the bacteria	MIC of usnic acid (*μ*g/mL)
A15	*Acinetobacter* spp.	>500
A25	*Acinetobacter* spp.	>500
A71	*Acinetobacter* spp.	>500
A72	*Acinetobacter* spp.	>500
A76	*Acinetobacter* spp.	>500
PA05	*Pseudomonas aeruginosa*	500
PA07	*Pseudomonas aeruginosa*	500
PA18	*Pseudomonas aeruginosa*	500
PA19	*Pseudomonas aeruginosa*	500
PA46	*Pseudomonas aeruginosa*	250
PA56	*Pseudomonas aeruginosa*	250
PA57	*Pseudomonas aeruginosa*	500
PA78	*Pseudomonas aeruginosa*	500
PA82	*Pseudomonas aeruginosa*	250

MIC: minimum inhibitory concentration.

**Table 3:** Evaluation of usnic acid and polymyxin B interaction by the Checkerboard method.

Clinical isolates	MIC (*μ*g/mL)		MIC of the combination (*μ*g/mL)	FICI	Interaction
	Polymyxin B	Usnic acid	Polymyxin B + usnic acid		
PA05	4	500	0.25/15.625	0.094	Synergism
PA07	4	500	0.25/15.625	0.094	Synergism
PA18	8	500	0.25/31.25	0.094	Synergism
PA19	4	500	0.5/15.625	0.156	Synergism
PA46	32	250	0.25/7.8125	0.039	Synergism
PA56	8	250	0.25/31.25	0.094	Synergism
PA57	32	500	0.25/15.625	0.039	Synergism
PA78	32	500	0.25/15.625	0.039	Synergism
PA82	4	250	0.25/15.625	0.125	Synergism

MIC: minimum inhibitory concentration; FICI: fractional inhibitory concentration index.

## Data Availability

The data of the present study can be acquired by contacting the corresponding author.
